# miR-125b and miR-223 Contribute to Inflammation by Targeting the Key Molecules of NFκB Pathway

**DOI:** 10.3389/fmed.2019.00313

**Published:** 2020-01-23

**Authors:** Swati Valmiki, Vineet Ahuja, Niti Puri, Jaishree Paul

**Affiliations:** ^1^School of Life Sciences, Jawaharlal Nehru University, New Delhi, India; ^2^Department of Gastroenterology, All India Institute of Medical Sciences, New Delhi, India

**Keywords:** microRNA, ulcerative colitis, TRAF6, A20, IKKα, NFκB pathway, inflammation

## Abstract

The contribution of miRNA in the pathogenesis of ulcerative colitis (UC) has emerged in the past few decades. Differential miRNA expression has been demonstrated in UC patients, and their ability to target the genes involved in inflammatory pathway has also been explored in recent years. miR-125b and miR-223 have been demonstrated to get upregulated within the colonic mucosa of UC patients. Here, we explored the biological relevance of miR-125b and miR-223 altered expression during UC by identifying the potential gene targets for miR-125b and miR-223. TRAF6 and A20, the signaling molecules involved in the NFκB pathway, were identified as target genes for miR-125b while IKKα was identified as a gene target for miR-223. The colonic mucosal samples from UC patients exhibited a significant rise in miR-125b and miR-223 expression while a subsequent downregulation was observed in the expression of TRAF6, A20, and IKKα. This negative correlation between miRNAs and their respective target genes was validated by co-transfecting miR-125b and miR-223 in HT29 cells. Co-transfection with miR-125b resulted in a marked decline in the expression of TRAF6 and A20, while the miR-223 co-transfected cells exhibited lower IKKα expression levels. Additionally, co-transfection with miR-125b or miR-223 in HT29 cells caused higher p65 and pro-inflammatory cytokines (IL-8 and IL-1β) expression upon LPS stimulation. From our findings, we highlight the possible contribution of miR-125b and miR-223 in regulating the inflammatory response during UC by negatively regulating the expression of TRAF6, A20, and IKKα. Therefore, we conclude that these two miRNAs could be considered as potential candidates for developing promising biomarkers for screening and diagnosis of UC.

## Introduction

Ulcerative colitis (UC) is a chronic, idiopathic, and relapsing inflammatory disorder of the gastrointestinal tract and is a major clinical subtype of inflammatory bowel disease (IBD). In UC, mucosal inflammation begins from rectum and progresses proximally in a continuous fashion affecting the gastrointestinal tract to a varying degree. During the diseased state, massive infiltration of neutrophils in the crypts and lamina propria is observed, which results in the formation of micro-abscesses. Patients diagnosed with UC are at higher risk of developing dysplasia and adenocarcinomas that often turn into colorectal cancer (CRC), compelling them to remain under lifelong regular endoscopic surveillance. The risk for CRC development depends on the disease duration and extent, like patients with pancolitis are more prone to develop CRC (1–4). Population-based studies indicated an increase in the IBD incidence in recent decades, among both adult and pediatric population, leading to manifold increase in global burden of IBD. Studies carried out in the northern part of India record an increase in the cases of UC with a prevalence rate varying from 42.8 to 44.3/10^5^ individuals, which is highest in the Asian subcontinent (5, 6).

The pathogenesis of IBD has not been completely understood to date, but the current understanding of IBD pathogenesis states that an altered immune response generated against the commensal flora of gut leads to IBD development under the influence of environmental factors (7). Despite our current understanding regarding the genetic basis of IBD, the monozygotic twin studies have suggested a concordance rate of 10–15% for UC and 30–35% for CD (8) while according to the GWAS, UC accounts only for 16% heritability (9). These reports leave gaps in our apprehension of IBD heritability but also highlight the contribution of environmental and epigenetic factors in IBD development and progression. The epigenetic factors, such as miRNA work at the interface of genetic and environmental factors; therefore, studying the association of miRNAs with the disease pathogenesis holds promise in the field of biomedical research. miRNAs have gained focus in the past few years due to their role in modulation of inflammatory response in various inflammatory and autoimmune disorders like multiple sclerosis, rheumatoid arthritis, inflammatory bowel disease, psoriasis, and various cancers (10–12). Over 2,600 miRNAs have now been identified that are functionally active. Each miRNA has the efficiency to bind and target multiple gene transcripts, and these miRNAs are members of complex gene regulatory networks (GRNs) (13). miRNAs actively regulate several biological processes, such as development, cell differentiation, proliferation, and apoptosis. Any alteration in miRNA expression could result in diminished cellular functioning and impaired downstream gene regulation, which implicates the role of miRNA in disease pathogenesis. Distinct miRNA expression has been reported in both the colonic mucosa and peripheral blood of IBD patients, and some of these miRNAs have been implicated to regulate the expression of genes involved in major inflammatory processes (14–16).

In the present study, we tried to explore the contribution of miR-125b and miR-223 in generating the inflammatory response during UC. Previously, microarray analyses carried out by our group observed altered miR-125b and miR-223 expression within the inflamed colonic mucosal regions of UC patients as compared to the non-IBD controls and non-inflamed colonic regions of UC patients (GEO accession number GSE99632) (17). Here, we first looked at the potential gene targets for these two miRNAs and then validated the microarray finding by performing qRT-PCR with increased sample size. The target gene expression was also investigated within the colonic mucosa of UC patients and the interaction between miR-125b and miR-223 with their respective gene targets was validated by performing *in vitro* studies. The biological relevance of miR-125b and miR-223 upregulation and their possible contribution to inflammation during UC was evaluated by looking at the expression of p65 subunit of NF-κB and its downstream pro-inflammatory cytokines in the presence and absence of miR-125b and miR-223 in LPS-stimulated HT29 cells.

## Methodology

### Sample Collection

Colonic mucosal pinch biopsies were collected from UC patients and non-IBD controls from the Department of Gastroenterology, All India Institute of Medical Sciences, New Delhi, India. The disease activity of UC patients was measured using the SCCAI score. The Simple Clinical Colitis Activity Index (SCCAI) score assesses the disease severity on the basis of disease symptoms, physical parameters, and sigmoidoscopic appearance. The parameters include bowel urgency, blood in stool, urgency of defecation, and extra colonic manifestations on the basis of which disease activity is scored as mild, moderate, or severe (18). The demographic features of patients have been enlisted in Table 1. Biopsies were collected from the colonic regions of patients showing clear symptoms of UC during colonoscopy, such as loss of vascular pattern, erythema, spontaneous bleeding, or ulceration. In case of control subjects, biopsies were collected from rectosigmoid area. Controls included were individuals attending the clinic for routine colonoscopy and were without any inflammatory disorder of intestine and without any IBD symptoms. The controls were age and sex matched with patients. All the biopsy samples were collected under the supervision of the gastroenterologist. The diagnosis of UC was confirmed with colonoscopic and histological examination following ECCO guidelines (19). The colonic pinch biopsies were collected in RNA later solution containing RNase inhibitors and transferred to JNU at 4°C and stored in −80°C deep freezer until processed further.

**Table 1:** Demographic features of study subjects.

**Characteristics**	**UC patients**	**Controls (*n*)**
No. of samples	48	30
Sex (M/F)	32/16	24/6
Age (years), mean ± SD (range)	37.02 ± 11.87 (20–65)	38.25 ± 13.91 (21–64)
Disease duration (years), mean ± SD (range)	4.906 ± 5.327 (1–26)	
**Disease extent**		
Proctitis	8 (16.67%)	
Proctosigmoiditis	12 (25%)	
Left-sided colitis	17 (35.42%)	
Pancolitis	11 (22.92%)	
**Medication**		
Mesalamine	31 (64.57%)	
Azathioprine	5 (10.42%)	
Steroids	0	0
**Smoking**		
Yes	5 (10.42%)	
No	43 (89.52%)	
Appendectomy (Y/N)	0/48 (0%)	

### Target Prediction for miR-125b and miR-223

Target prediction for miR-125b and miR-223 was carried out by miRWalk database (http://www.ma.uniheidelberg.de/apps/zmf/mirwalk/), which simultaneously searches several other databases for predicting the potential gene targets for miRNAs (20) Additionally, TargetScan, MIRDB, mirna.org, PicTar, DIANA-MicroT-CDS, RNA22, and MirTarBase were also employed for target prediction, and the cases where the same gene was picked by at least three tools were chosen as a potential gene target. The involvement of target genes in different biological pathways was studied with miRPath v.3: DIANA TOOLS.

### Reverse Transcription and qRT-PCR

Total RNA was isolated from the mucosal biopsies obtained from UC patients and non-IBD controls using mirVana miRNA isolation kit (Ambion Inc., TX, USA) following the manufacturer's protocol. RNA quality and quantity were assessed by NanoDrop 2000 Spectrophotometer (Thermo Scientific, Waltham, USA). miRNAs and their respective target genes were reverse transcribed using revert aid cDNA synthesis kit (Fermentas St. Leon Rot, Germany). miR-125b and miR-223 were reverse transcribed using gene-specific stem-loop primers (21) while the target genes TRAF6, A20, and IKKα were reverse transcribed with a random hexamer primer. Differential expression of miRNAs and their respective target genes was investigated by performing qRT-PCR using BioRad CFX96 Real Time System with C1000 Touch Thermalcylcer (BioRad, Hercules, CA, USA) by SYBR Green method. The relative expressions of miRNAs and target genes were normalized with internal reference U6 snoRNA and GAPDH, respectively, and analyzed using 2^−ΔΔ*ct*^ method. The details of primers used for reverse transcription and qRT-PCR are given in Table 2.

**Table 2:** Primers used for reverse transcription (RT) and qRT-PCR for miRNAs and qRT-PCR for target genes.

**Name**	**Primer**	**Sequence (5^′^-3^′^)**
Reverse universal primer		GTGCAGGGTCCGAGGT
hsa-miR-125b-5p	RT	GTCGTATCCAGTGCAGGGTCCGAGGTA TTCGCACTGGATACGTCACAAGT
	Forward	TCCCTGAGACCCTAACTTG
hsa-miR-223-3p	RT	GTCGTATCCAGTGCAGGGTCCGAGG TATTCGCACTGGATACGTGGGGTAT
	Forward	TGTCAGTTTGTCAAATACCC
Sno RNA U6	RT	GTCGTATCCAGTGCAGGGTCCGAGGT ATTCGCACTGGATACGAAAATATG
	Forward	CAAATTCGTGAAGCGTTCCA
STAT3	Forward	GGCTTTTGTCAGCGATGGAG
	Reverse	ATTTGTTGACGGGTCTGAAG
TRAF6	Forward	CCTTTGGCAAATGTCATCTGTG
	Reverse	CCTTTGGCAAATGTCATCTGTG
IKKα	Forward	CGGCTTCGGGAACGTCTG
	Reverse	GCCTTTACAACATTGGCATGG
GAPDH	Forward	GCTCCTCCTGTTCGACAGTCA
	Reverse	GCAACAATATCCACTTACCAG

### Cloning of miRNAs and Their Gene Targets

In order to construct the plasmids expressing miR-125b and miR-223, we amplified the DNA fragment containing pre-miR-125b and pre-miR-223 from human genomic DNA using PCR primers with EcoR1 site inserted at the 5′ end of forward primer and BamH1 at the 5′ end of reverse primer. The amplified fragments were cloned into pBABE-puro retroviral vectors within BamH1 and EcoR1 sites.

Similarly, the 3′UTR regions of target genes bearing the specific binding site for their respective miRNAs were amplified from human genomic DNA using PCR primers with Sac1 restriction site inserted at the 5′ end of forward primer and Mlu1 restriction site inserted at the 5′ end of reverse primer. The amplified fragments were then cloned into pMIR-REPORT-miRNA Expression Reporter Vector, downstream the luciferase gene within the Sac1 and Mlu1 restriction sites. Details of primers used for miRNA and target gene cloning are given in Table 3.

**Table 3:** Primers used for cloning of miRNAs and their respective gene targets.

**Name**	**Primer**	**Sequence**
miR-125b	Cloning F.P	CGAGCTCCCTCTCCTACCAAGCAG
miR-125b	Cloning R.P	GACGCGTGTCCATGGATGGTTCTG
miR-223	Cloning F.P	CGCGGATCCCTTCAGGATCTCTCTTCTGG
miR-223	Cloning R.P	CCGGAATTCCCCTGGTGTCCTCAGAT
TRAF6	Cloning F.P	CGAGCTCGCTAGGATTAGAAGTCACAGTG
TRAF6	Cloning R.P	GACGCGTCTCTGATTTGGCCTCTCTG
IKKα	Cloning F.P	CGAGCTCGGGATTGTGTATCTGTGCTTC
IKKα	Cloning R.P	GACGCGTCGATGATAGAGGTCCACAGTCC
A20	Cloning F.P	CGAGCTCCCTCTCCTACCAAGCAG
A20	Cloning R.P	GACGCGTGTCCATGGATGGTTCTG

### Construction of miR-223 and miR-125b Mutants

The pBABE-puro vectors having a confirmed miR-125b or miR-223 insert were used as a template to generate the mutants for miR-125b and miR-223. Three nucleotides within the seed region of miRNA were substituted in order to restrict their binding with the 3′UTR of target genes. The site-directed mutagenesis was carried out with QuickChange^®^ II XL Site-Directed Mutagenesis Kit (Stratagene) following the manufacturer's protocol. Primers used for the generation of site-directed mutants for miR-125b and miR-223 are given in Table 4.

**Table 4:** Primer sequences used for site-directed mutagenesis of miR-125b and miR-223.

**Name**	**Primer**	**Sequence**
miR-125b	F.P	CGCTCCTCTCAGTCAAGGAGACCCTAAC
miR-125b	R.P	GTTAGGGTCTCCTTGACTGAGAGGAGCG
miR-223	F.P	CACTCCATGTGGTAGAGTGTACTTTTGTCAAATACCCC
miR-223	R.P	GGGGTATTTGACAAAAGTACACTCTACCACATGGAGTG

### Cell Culture Conditions

HT29 human colon adenocarcinoma cell line was cultured in Dulbecco's Modified Eagle Medium with high glucose (DMEM) (Gibco, Invitrogen) supplemented with 5% heat-inactivated fetal bovine serum (FBS) and 5% Penicillin–Streptomycin (PenStrep) (Thermo Fisher Scientific). Cells were incubated at 37°C and 5% CO_2_, and culture media was changed 2–3 times a week. Cells were passaged regularly and when a confluency of 90% was reached.

### Gene Expression Assay

HT29 cells were seeded in 12-well plates to attain a confluency of 70–90%. Next day, the respective pBABE-puro vector with miRNA insert and pMIR-REPORT with target gene 3′UTR insert were co-transfected into the HT29 cells using Lipofectamine 2000 (Thermo Fisher Scientific, USA) following the manufacturer's protocol. An empty pBABE-puro vector without miRNA insert and untransfected HT29 cells were used as positive controls while the pBABE-puro vectors with mutated miRNA insert were used as the negative control. The expression levels of probable target genes like TRAF6, A20, and IKKα were investigated by performing qRT-PCR, 48 h post-transfection.

### Dual Luciferase Reporter Assay

HT29 cells were seeded in 96-well plates to attain a confluency of 70–90%. The next day, pBABE-puro vector with miRNA insert and pMIR-REPORT with 3′UTR of the target gene were co-transfected to the cells along with transfection control Renilla luciferase expression construct pRL-TK (Promega) using Lipofectamine 2000 (Thermo Fisher Scientific, USA) using manufacturer's protocol. An empty pBABE-puro vector without miRNA insert and untransfected HT29 cells were used as positive controls and pBABE-puro vector with mutant miRNA insert was used as negative control. The Luciferase assay was performed 24 h post-transfection using Dual Luciferase Reporter Assay System (Promega). The activity of firefly luciferase was normalized with Renilla luciferase for each sample.

### Lipopolysaccharide (LPS) Stimulation and NFκB Activation

To study the impact of miRNA and target gene interaction on downstream NFκB signaling, we co-transfected the plasmid constructs pBABE-puro-miRNA and pMIR-REPORT-3′UTR in HT29 cells. After 48 h post-transfection, the HT29 cells were stimulated with 100 ng/ml of LPS for 6 h to activate the downstream inflammatory pathway. After 6 h of stimulation, total RNA was isolated from the cells and expression levels of p65 and its downstream pro-inflammatory cytokines, IL-8 and Il-1β, were by measured by qRT-PCR. An empty pBABE-puro vector without miRNA insert was used as positive control, and pBABE-puro with mutant miRNA insert was used as negative control.

### Statistical Analysis

The statistical analysis was done using unpaired, Student's *t*-test where the mean of two independent groups is compared and a *p* ≤ 0.05 was considered significant. The error bars in all the graphs represent standard error of the mean (SEM).

## Results

### miR-125b and miR-223 Target Inflammatory Genes

**Figure 1:**
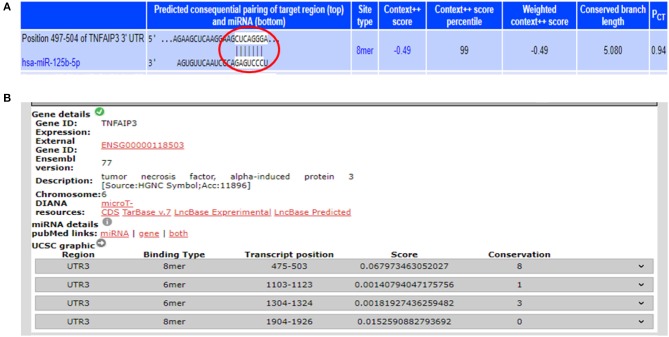
Binding sites in 3′UTR of A20 for miR-125b. **(A)** 3′UTR of A20 contains binding sites for miR-125b-5p at position 497–504 as shown by target scan (http://www.targetscan.org/cgi-bin/targetscan/vert_72/view_gene.cgi?rs=ENST00000237289.4&taxid=9606&showcnc=0&shownc=0&shownc_nc=&showncf1=&showncf2=&subset=1#miR-125-5p). **(B)** miR-125b binding sites within the 3′UTR of A20 were also shown by DIANA-microTCDS (http://diana.imis.athena-innovation.gr/DianaTools/index.php?r=microT_CDS/results&keywords=ENSG00000118503&genes=ENSG00000118503%20&mirnas=&descr=&threshold=0.7).

**Figure 2:**
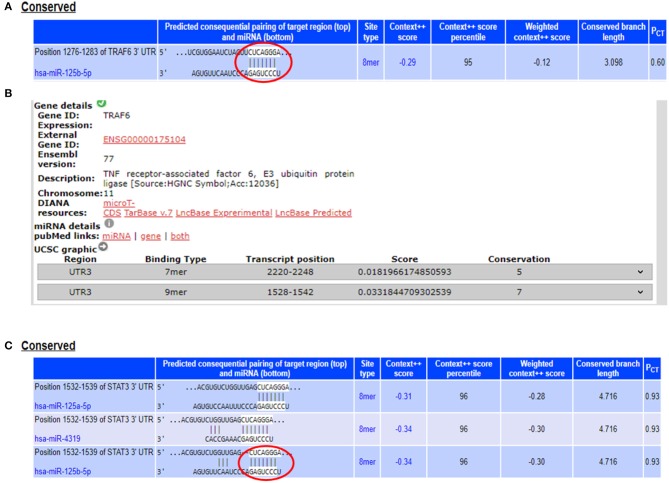
miR-125b binding sites within the 3′UTRs of TRAF6 and STAT3. **(A)** 3′UTR of TRAF6 contains binding sites for miR-125b-5p at position 1276–1283 as shown by target scan (http://www.targetscan.org/cgi-bin/targetscan/vert_72/view_gene.cgi?rs=ENST00000526995.1&taxid=9606&showcnc=0&shownc=0&shownc_nc=&showncf1=&showncf2=&subset=1#miR-125-5p). **(B)** miR-125b binding sites within the 3′UTR of TRAF6 were also shown by DIANA-microT-CDS (http://diana.imis.athena-innovation.gr/DianaTools/index.php?r=microT_CDS/results&keywords=ENSG00000175104&genes=ENSG00000175104%20&mirnas=&descr=&threshold=0.7). **(C)** 3′UTR of STAT3 contains binding sites for miR-125b-5p at position 1532–1539 as shown by target scan (http://www.targetscan.org/cgi-bin/targetscan/vert_72/view_gene.cgi?rs=ENST00000585517.1&taxid=9606&members=&showcnc=0&shownc=0&showncf1=&showncf2=&subset=1#miR-125-5p).

From the miRNA target prediction tools, we identified TRAF6 (TNF receptor associated factor 6), TNFAIP3 (TNF alpha induced protein 3) also referred to as A20, STAT3 (signal transducer and activator of transcription 3), TP53, and IL6R (Interleukin 6 receptor) as potential gene targets for miR-125b. A20 was picked up as a target of miR-125b by three target prediction tools: TargetScan, DIANA-microT-Tarbase, and miRNA.org. TargetScan and DIANA microT-CDS revealed an 8 mer site in the 3′UTR of A20 at transcript position 497–504 targeted by miR-125b (Figure 1). TRAF6 was also picked up as a target by three prediction tools: TargetScan, DIANA-microT-CDS, and miRNA.org. It also bears an 8mer target site for miR-125b in its 3′UTR at transcript position 1276–1283 as shown by TargetScan (Figure 2). Both TRAF6 and A20 are key molecules involved in the canonical NFκB pathway and a hyperactive NFκB is reported in the macrophages and epithelial cells derived from inflamed intestinal mucosa (22). Therefore, we selected these two genes as potential targets for miR-125b. Further, STAT3 was picked up as a target by three bioinformatics tools TargetScan, MIRDB, and DIANA-microT-CDS, bearing an 8mer binding site for miR-125b at transcript position 1532–1539 (Figure 2). STAT3 is known to act as a positive regulator for Th17 cell differentiation, and during UC, an exaggerated Th17 response leading to overproduction of Th17 derived cytokine is observed (23, 24).

**Figure 3:**
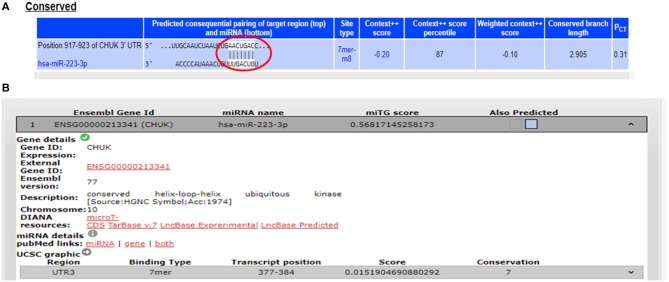
Binding sites in 3′UTR of IKKα/CHUK for miR-223. **(A)** 3′UTR of IKKα contains 7mer-m8 binding sites for miR-223 at position 917–923 as shown by target scan. **(B)** miR-223 binding site within the 3′UTR of IKKα was also shown by DIANA-microT-CDS.

The potential gene targets for miR-223 included TAB3 (TGF beta associated kinase 3), ICAM1 (Intercellular Cell Adhesion Molecule 1), and IKKα (IkB Kinase alpha), and all of these three genes are shown to be involved in intestinal inflammation. ICAM1, a cell adhesion molecule, was identified as miR-223 target by DIANA-microT-CDS and miRNA.org, while TAB3, a signaling molecule involved in NFκB pathway, was identified as miR-223 target only by DIANA-microT-CDS. TargetScan, miRNA.org, and DIANA-mirTarBase displayed IKKα as a gene target for miR-223. According to TargetScan, IKKα showed a 7mer-m8 site in its 3′UTR for miR-223 at transcript position 917–923 (Figure 3). We selected IKKα as a potential gene target for miR-223. It is generally perceived that IKKα plays a major role in regulating non-canonical NFκB pathway, which is primarily dedicated to B cell maturation and lymphoid organogenesis, but it is also responsible for resolving the acute inflammatory response by terminating the pro-inflammatory cytokine signaling generated due to prolonged NFκB activation (25).

### The Target Genes Exhibited an Inverse Pattern of Expression With Respect to Their Specific miRNAs in Patient vs. Control Samples

**Figure 4:**
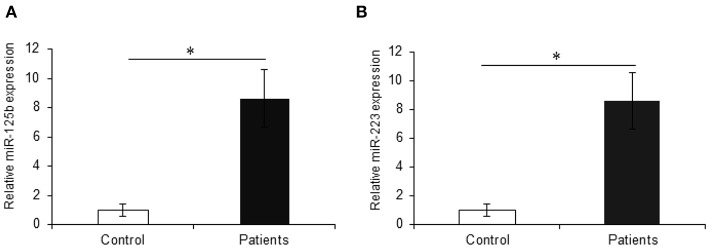
Relative expression of miR-125b and miR-223 in UC patients. qRT-PCR for miR-125b and miR-223 was performed in UC vs. control (*n* = 20 for non-IBD controls and *n* = 30 for active UC patients). **(A)** miR-125b exhibited significantly higher expression in UC patients as compared to controls with a change of around 9.85-fold. **(B)** miR-223 showed significantly higher (8.63-fold) expression in UC patients as compared to controls. U6 was used as internal reference. ^*^ Denotes the level of significance. ^*^p ≤ 0.05. Bars represent standard error of the mean (SEM).

The earlier microarray findings were validated by performing qRT-PCR for miR-125b and miR-223 with increased sample size including 30 active UC patients and 20 non-IBD controls. In accordance with the microarray results, the qRT-PCR analysis showed a significant upregulation in the expression of miR-125b in UC patients as compared to non-IBD controls. For miR-125b, we observed a 9.85-fold higher expression in UC patients as compared to the non-IBD controls (*p* = 0.004). Similarly, miR-223 showed a significant upregulation in UC patients as compared to the non-IBD controls with a fold change of 8.63 (*p* = 0.012) (Figure 4).

**Figure 5:**
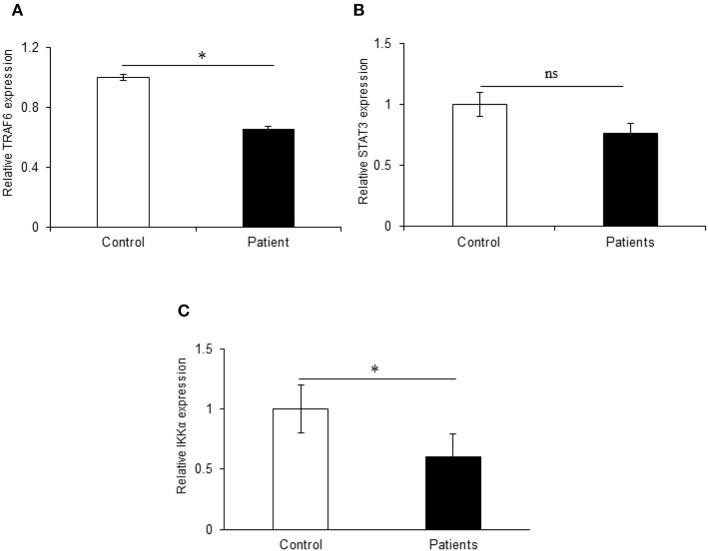
Relative expression of target genes in UC patients. qRT-PCR for target genes was performed in 20 controls and 30 IBD patients. **(A)** TRAF6 exhibited significant 1.47-fold upregulation in UC patients as compared to non-IBD controls. **(B)** STAT3 did not show significant changes in expression in UC patients as compared to controls. **(C)** IKKα showed significant 0.40-fold downregulation in UC patients as compared to controls. GAPDH gene was used as internal reference. ^*^ Denotes the level of significance. ^*^p ≤ 0.05. ns, Not significant. Bars represent standard error of the mean (SEM).

We also investigated the expression of TRAF6 and STAT3 that are potential targets of miR-125b. TRAF6, a key signaling molecule in the canonical NFκB pathway, exhibited a significant downregulation with a fold change of 0.35 in UC patients as compared to non-IBD controls (*p* = 0.03) showing an inverse relation with the miR-125b expression. We observed a downregulation in the expression of STAT3 in UC patients with 0.25-fold change as compared to non-IBD controls but the change was not significant (*p* = 0.62). With the same set of samples, our earlier reports have indicated decreased expression of A20 in UC patients at the protein level (26). IKKα, a potential target for miR-223, showed a significant 0.40-fold downregulation in UC patients as compared to non-IBD controls. This proves an inverse correlation of IKKα expression with the increased expression of miR-223 (*p* = 0.014) (Figure 5).

### *In vitro* Assay Shows That miR-125b Negatively Regulates the Expression of A20 and TRAF6

**Figure 6:**
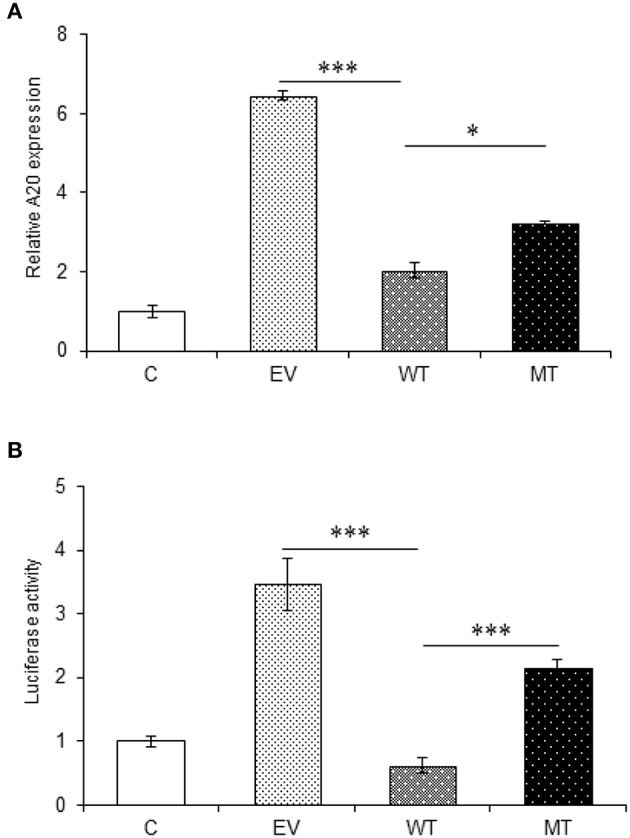
A20 expression in the presence of miR-125b. A20 exhibited decreased expression in the presence of miR-125b **(A)** miR-125b and A20 were co-transfected in HT29 cells for 48 h. After 48 h, RNA was isolated and reverse transcribed to investigate the mRNA expression of A20 through qRT-PCR. GAPDH gene was used as an internal reference. **(B)** miR-125b and A20 were co-transfected in HT29 cells along with pRL-TK control plasmid for 24 h. After 24 h, the dual luciferase reporter assay was performed to evaluate the luciferase activity corresponding to A20 expression. ^*^ Denotes level of significance. ^*^p ≤ 0.05, ^***^p ≤ 0.001. C, untransfected control; EV, empty vector; WT, wild type; MT, mutant. Bars represent standard error of the mean (SEM).

The miRNA target prediction tools predicted putative binding sites for miR-125b within the 3′UTR of TRAF6 and A20 gene. The gene expression assay showed a significant decrease in A20 expression in the HT29 cells co-transfected with wild type miR-125b with a fold change of 4.41 as compared to the empty vector control (*p* > 0.0001). This reduced A20 expression was restored to a significant level when the mutated form of miR-125b bearing mutation in the seed region was present (*p* = 0.02). The results obtained from gene expression assay were further confirmed by performing a dual luciferase reporter assay, which is a high-throughput method to study the gene expression at transcriptional levels. It allows the quantitation of both Firefly and Renilla luciferases from a single sample in mammalian cell culture. In the dual luciferase reporter assay, the relative Renilla luciferase activity was 2.84-fold lower in the presence of wild-type miR-125b as compared to the empty vector control (*p* = 0.0002) that correlated with the A20 expression in the presence of miR-125b. In the presence of mutated miR-125b, the Renilla luciferase activity again increased to a significant level (1.52-fold increase) as compared to the wild miR-125b (*p* > 0.0001) (Figure 6).

**Figure 7:**
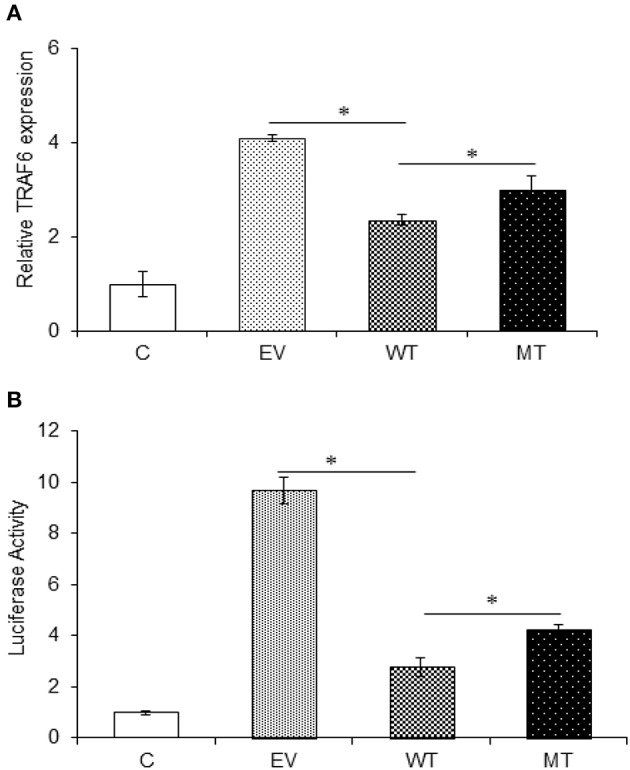
TRAF6 expression in presence of miR-125b. A decreased TRAF6 expression was observed in the presence of miR-125b in HT29 cells. **(A)** For gene expression assay, miR-125b and TRAF6 were co-transfected in HT29 cells for 48 h. After 48 h, RNA was isolated and reverse transcribed to investigate the mRNA expression of TRAF6 through qRT-PCR. GAPDH gene was used as an internal reference. **(B)** For dual luciferase reporter assay, miR-125b and TRAF6 were co-transfected in HT29 cells for 24 h along with pRL-TK control plasmid, which was used as internal reference. After 24 h, the dual luciferase reporter assay was performed to evaluate the luciferase activity, which corresponds to TRAF6 expression. *P* < 0.05 were statistically significant. ^*^p ≤ 0.05. C, untransfected control; EV, empty vector; WT, wild type; MT, mutant. Bars represent standard error of the mean (SEM).

The interaction between miR-125b and TRAF6 was validated by following the same protocol as miR-125b and A20. In the gene expression assay, the co-transfection with the wild type miR-125b potentially reduced the expression of TRAF6 to a significant level by 1.74-fold with respect to the empty vector control (*p* = 0.0152), while in the presence of mutated miR-125b, the TRAF6 expression was restored to a significant level (0.65-fold) as compared to the wild-type miR-125b (*p* = 0.0225). Similarly, in the dual luciferase reporter assay, the luciferase activity that corresponds to the expression of TRAF6 significantly decreased in the presence of wild-type miR-125b by 6.95-fold (*p* = 0.0032), which again showed significant increased expression in the presence of mutated miR-125b by 1.47-fold (*p* = 0.0187) (Figure 7).

### p65 Subunit of NFκB and Pro-inflammatory Cytokines Were Highly Upregulated in the Absence of TRAF6 and A20

**Figure 8:**
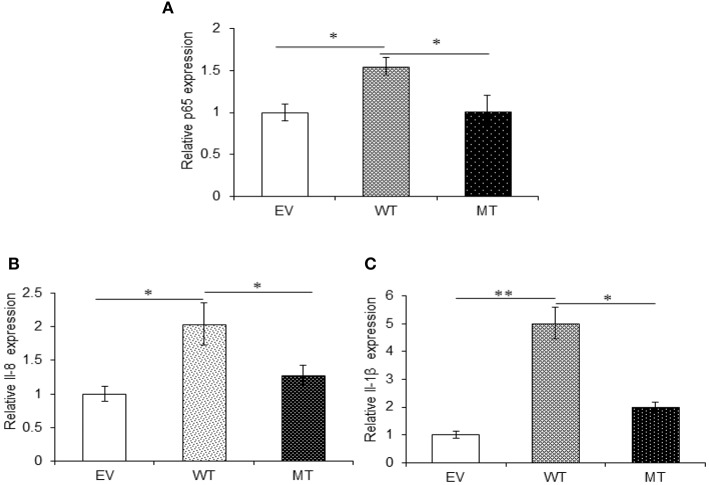
A20 absence results in an increased pro-inflammatory response. miR-125b and A20 were co-transfected in HT29 cells for 48 h and stimulated with 100 ng/ml of LPS. After 6 h of stimulation, RNA was isolated and reverse transcribed to investigate the mRNA expression of p65, IL-8, and IL-1β through qRT-PCR. p65 **(A)**, IL-8 **(B)**, and IL-1β **(C)** showed significant rise in their expression in the miR-125b overexpressed cells where it suppressed the expression of A20. While in the presence of mutant miR-125b, their expression showed a marked decrease. GAPDH gene was used as an internal reference. *P* < 0.05 were statistically significant. Significance was estimated with respect to the pBABE-puro empty vector control group. ^*^p ≤ 0.05, ^**^p ≤ 0.01. EV, empty vector; WT, wild type; MT, mutant. Bars represent standard error of the mean (SEM).

Both A20 and TRAF6 exhibited a marked decrease in their expression in UC patients and we have shown that miR-125b efficiently targets the 3′UTR and negatively regulates their expression. Next, to find the biological relevance of miR-125b and TRAF6/A20 interaction in generating inflammatory response, we evaluated the effect of miR-125b-mediated downregulation of TRAF6 and A20 on downstream inflammatory response generated through NFκB signaling. This was performed by co-transfecting the HT29 cells with miR-125b along with TRAF6 or A20-3′UTR and stimulating the cells with LPS followed by investigation of p65 and pro-inflammatory expression by qRT-PCR. A20 acts as a feedback regulator in the NFκB pathway where it negatively regulates the NFκB activation to resolve inflammation (27). We expected an increased expression of p65 and pro-inflammatory cytokines in the absence of A20. In the presence of wild-type miR-125b where it could target the expression of A20, we observed a 1.55-fold higher p65 expression as compared to the empty vector control (*p* = 0.03). The increased p65 expression was again significantly suppressed in the presence of mutant miR-125b where it was no longer able to target A20 (*p* = 0.03). Similar to p65, IL-8 expression was increased to a significant level (2.0-fold) in the presence of miR-125b, where it inhibited the expression of A20 (*p* = 0.019). In the presence of mutant miR-125b, the IL-8 expression was again decreased to a significant level where A20 was able to carry out its negative regulation (*p* = 0.04). Similarly, IL-1β exhibited significant upregulation in the presence of miR-125b where it inhibited the expression of A20 with a fold change of 5.02 (*p* = 0.0019). However, in the presence of mutant miR-125b, we observed 3.01-fold reduced IL-1β expression (*p* = 0.04) probably due to the regulatory effect of A20 (Figure 8).

**Figure 9:**
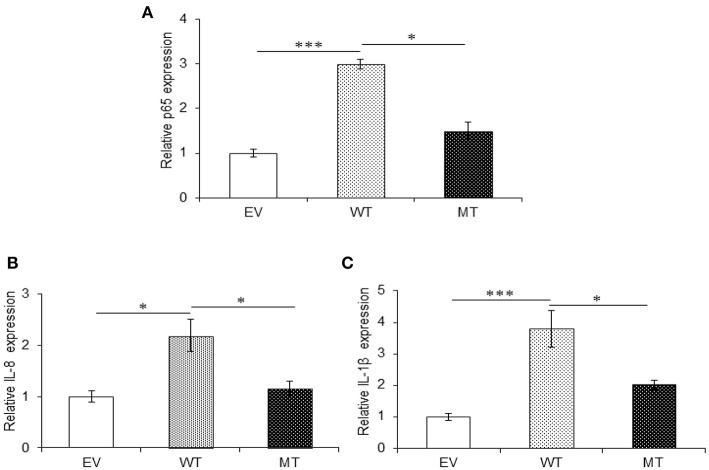
Absence of TRAF6 results in higher pro-inflammatory response. miR-125b and TRAF6 were co-transfected in HT29 cells for 48 h and stimulated with 100 ng/ml of LPS. After 6 h of stimulation, RNA was isolated and reverse transcribed to investigate the mRNA expression of p65, IL-8, and IL-1β through qRT-PCR. p65 **(A)**, IL-8 **(B)**, and IL-1β **(C)** showed significant higher expression in cells transfected with miR-125b, which negatively regulates the expression of TRAF6 gene while the presence of mutated form of miR-125b resulted in marked decrease in their expression. GAPDH gene was used as an internal reference. *P* < 0.05 were statistically significant. Significance was estimated with respect to the pBABE-puro empty vector control group. ^*^p ≤ 0.05, ^***^p ≤ 0.001. Bars represent standard error of the mean (SEM).

Similar to A20, in the presence of wild-type miR-125b where it could target the expression of TRAF6, we observed 3.0-fold higher p65 expression (*p* = 0.0004), while in the presence of the mutated form of miR-125b, p65 expression was suppressed to a significant level with 1.5-fold change (*p* = 0.047). The expression of both IL-8 (*p* = 0.047) and IL-1β (0.0003) significantly increased in the presence of miR125b where it negatively regulates the expression of TRAF6. Similarly, as observed with p65, in the presence of mutated miR-125b, IL-8 (*p* = 0.0354), and IL-1β (0.0326) showed decreased expression by 1.25- and 1.78-fold, respectively (Figure 9).

### miR-223 Binds Within the 3′UTR of IKKα Gene and Negatively Regulates Its Expression

**Figure 10:**
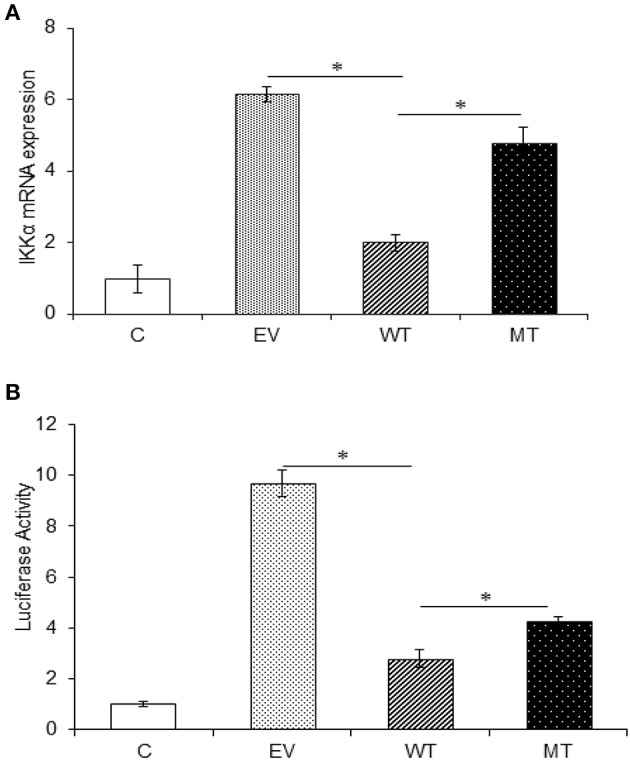
IKKα expression is negatively regulated by miR-223. IKKα showed a decreased expression in the presence of miR-223, which was again increased to a significant point in the presence of mutated form of miR-223. **(A)** miR-223 and IKKα were co-transfected in HT29 cells for 48 h. After 48 h, RNA was isolated and reverse transcribed to investigate the mRNA expression of IKKα through qRT-PCR. GAPDH gene was used as an internal reference. **(B)** miR-223 and IKKα were co-transfected in HT29 cells along with pRL-TK control plasmid, which was used as internal reference, for 24 h. After 24 h, the dual luciferase reporter assay was performed to access the luciferase activity corresponding to IKKα expression. *P* < 0.05 were statistically significant. ^*^ Denotes level of significance. ^*^p ≤ 0.05. C, untransfected control; EV, empty vector; WT, wild type; MT, mutant. Bars represent standard error of the mean (SEM).

In the gene expression assay, we observed a 4.16-fold decrease in IKKα expression in the HT29 cells co-transfected with the wild-type miR-223 as compared to the empty vector control. While in the presence of mutated miR-223 bearing mutation in the seed region, the IKKα expression again showed a marked increase (2.78-fold) (*p* = 0.02). Similarly, in the dual luciferase reporter assay, we observed significant reduction in Renilla luciferase expression to about 2.78-fold, in the presence of miR-223 that corresponds to the lower IKKα expression (*p* = 0.0004). Again in the presence of mutant miR-223 where it was no longer able to target IKKα, the Renilla luciferase expression was increased to a significant level (*p* = 0.0007) (2.0-fold) (Figure 10).

### p65 Subunit of NFκB and Pro-inflammatory Cytokines Showed Decreased Expression in the Absence of IKKα

**Figure 11:**
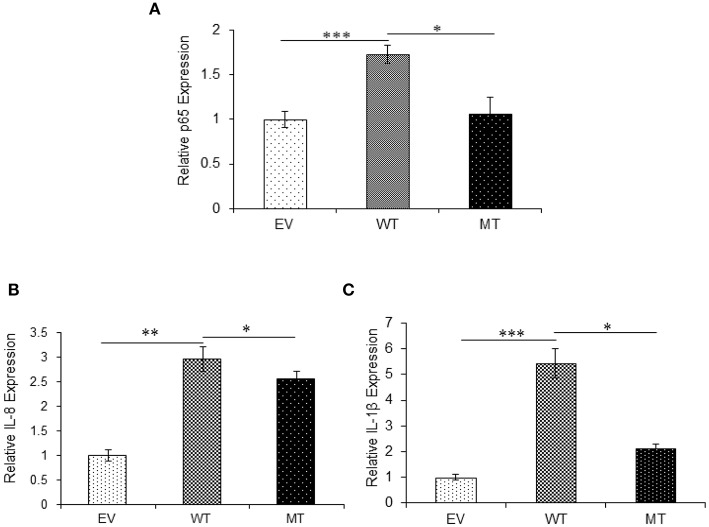
IKKα absence results in higher pro-inflammatory response. p65 and its downstream pro-inflammatory cytokines showed significant higher expression in HT29 cells in the presence of miR-223 while a decrease in the presence of mutated form of miR-223. miR-223 and IKKα were co-transfected in HT29 cells for 48 h and stimulated with 100 ng/ml of LPS. After 6 h of stimulation, RNA was isolated and reverse transcribed to investigate the mRNA expression of **(A)** p65, **(B)** IL-8, and **(C)** IL-1β through qRT-PCR. GAPDH gene was used as an internal reference. *P* < 0.05 were statistically significant. Significance was estimated with respect to the pBABE-puro empty vector control group. ^*^p ≤ 0.05, ^**^p ≤ 0.01, ^***^p ≤ 0.001. EV, empty vector; WT, wild type; MT, mutant. Bars represent standard error of the mean (SEM).

The upregulated miR-223 and downregulated expression of IKKα could be one of the contributing factors to chronic inflammation observed during UC. Since NFκB is one of the major inflammatory pathways, we investigated the effect of miR-223 and IKKα interaction on the downstream inflammatory response by looking at p65 and pro-inflammatory cytokine expression levels. We observed significant 1.73-fold higher p65 expression in the presence of wild-type miR-223, where it blocked the expression of IKKα as compared to the empty vector control (*p* = 0.0001). While in the presence of mutated miR-223, which is now unable to target IKKα, we observed a decreased p65 expression (1.06-fold) to a significant level (*p* = 0.028). Similar to the trend of p65 expression, we observed an increased IL-8 (*p* = 0.0023) and IL-1β (*p* = 0.0005) expression in the presence of wild-type miR-223 with a fold change of 2.97 and 5.43, respectively. In the presence of mutated miR-223, both IL-8 (*p* = 0.037) and IL-1β (0.0418) exhibited significant decreased expression with a change of 2.57- and 2.13-fold, respectively (Figure 11).

## Discussion

Our previous microarray analysis revealed that the miRNA signatures within the inflamed colonic region of UC patients differ significantly from the non-inflamed ones as well as from the non-IBD controls (17). Among the miRNAs that showed upregulated expression, we considered miR-125b and miR-223 as two very important miRNA because upregulation of these two miRNA has previously been reported in UC patients; however, their target genes had not been identified with context to UC (28). miR-125b was found to be directly associated with cancer progression and poor prognosis of CRC in patients, suggesting that miR-125b could prove as a prognostic marker for CRC (29). Similarly, miR-223 has also been identified to play a crucial role in regulating the differentiation of intestinal dendritic cells and macrophages that are required to maintain the intestinal homeostasis, and miR-223 is known to exert these regulatory effects by directly targeting the C/EBPβ gene (30). Recently, the oncogenic properties of miR-223 during CRC have also been reported where higher expression of miR-223 enhanced the cancer progression by targeting p120 catenin (31). The qRT-PCR results for miR-125b validated our microarray results as its expression was higher in UC patients as compared to non-IBD controls. Our qRT-PCR findings for miR-125b supported the previous report where increased miR-125b expression was seen in UC patients, and it was also proposed as an important diagnostic marker for UC (32). Higher miR-125b expressions are also seen in tissues obtained from gastric cancer patients as compared to non-cancerous gastric tissues where higher miR-125b levels lead to gastric cancer progression. The upregulation of miR-125b was also found to be associated with poor prognosis and trastuzumab resistance in HER-2-positive gastric cancer patients (33).

NFκB signaling plays a pivotal role in mediating a successful immune response, but the inflammatory response generated through this signaling pathway should not last longer as it may result in tissue damage and death. Prolonged activation of the NFκB pathway is one of the leading causes of several autoimmune diseases including IBD and various cancers (34–36). TRAF6 and A20 are the two key signaling molecules involved in NFκB pathway, and both these genes bear complementary binding sites for miR-125b in their 3′UTRs. Therefore, these two genes were selected as potential gene targets for miR-125b for assessing their possible role in mediating inflammatory response during UC. TRAF6 is an E3 ubiquitin ligase, which is essential for the synthesis of “Lys-63”-linked-polyubiquitin chains, which are conjugated to different proteins with the help of UBE2N and UBE2V1. TRAF6 is a key molecule in the activation of NFκB pathway as the synthesis of Lys-63 linked auto-polyubiquitin chains is responsible for the activation of downstream pathway, but the exact mechanism by which the Lys-63 polyubiquitin chains facilitate the activation of signaling pathway remains elusive. The contribution of TRAF6 in colitis development has been explored earlier, but the results are not conclusive. While one study reports the colitis development in mice due to deletion of TRAF6 from intestinal epithelial cells, another study reports a higher TRAF6 expression in the colonic mucosa of UC and CD patients as compared to the non-IBD individuals (37, 38).

The UC patients included in our study showed a significantly reduced expression of TRAF6 in their colonic mucosa with respect to the non-IBD controls, as opposed to miR-125b, which showed significant higher expression in UC patients. The validation of miR-125b and TRAF6 interaction was performed in HT29 cells where the reduced TRAF6 expression in the HT29 cells co-transfected with the wild-type miR-125b as compared to cells co-transfected with empty pBABE vectors indicated that miR-125b binds within the 3′UTR of TRAF6 and negatively regulates its expression. The interaction between miR-125b and TRAF6 gets hampered when a mutation is present within the seed region of miR-125b as we observed a partial restoration of TRAF6 expression in the cells transfected with mutant miR-125b with respect to the wild-type miR-125b. This observation was consistent with a previous study where TRAF6 was proved as a potential gene target for miR-125b in human osteoarthritic chondrocytes (39). Our findings from *in vitro* studies confirmed that TRAF6 is a potential gene target for miR-125b, and its expression is negatively regulated by miR-125b. While looking at the NFκB mediated inflammatory response generated in the presence and absence of TRAF6, we found significant increase in the expression of p65 and pro-inflammatory cytokines (IL-8 and IL-1β) in HT29 cells co-transfected with wild-type miR-125b, which can efficiently suppress the expression of TRAF6 gene, while a mutation in miR-125b seed region that hampers the binding affinity of miR-125b resulted in further decline in the p65 and pro-inflammatory cytokines expression. Here, we highlight that besides the E3 ubiquitin ligase activity, TRAF6 might have an additional regulatory role in the NFκB signaling, and based on the findings obtained from our study, TRAF6 seems to play a role in the negative regulation of inflammatory response as suppression of its expression by miR-125b resulted in higher inflammatory response. We also suggest that the upregulated miR-125b expression in the UC patients might be responsible for the downregulated TRAF6 expression and this miR-125b-mediated suppression of TRAF6 gene could be one of the contributory factors to the chronic inflammation observed during UC. The role of TRAF6 in mediating negative regulation of the NFκB pathway has not been reported to date and further detailed studies are needed to understand at what point in the pathway of NFκB activation TRAF6 acts and how it regulates the inflammatory response.

A20, which is a well-known regulator of the NFκB pathway, was selected as another probable target for miR-125b (40). Decreased A20 expression has been reported from pediatric UC patients, and its role in regulating the inflammatory response has been explored extensively (41, 42). In a previous study conducted by our group, reduced A20 protein levels were observed in the colonic mucosal samples from UC patients as compared to the non-IBD controls. The A20 mRNA levels were reported to be higher in UC patients in this study, and this could be due to the presence of miR-125b binding sites in its 3′UTR and miR-125b probably regulates its expression through translational inhibition. Therefore, we directly validated the miR-125b and A20 interaction through *in vitro* studies (26). We observed a marked decrease in A20 expression in the HT29 cells co-transfected with the wild-type miR-125b as compared to the empty vector control, which again increased to a significant level in the cells co-transfected with the mutant miR-125b bearing mutation in its seed region. This confirmed that miR-125b can efficiently bind within the 3′UTR of A20 gene and negatively regulate its expression. While looking at the inflammatory response generated in the absence and presence of A20, we observed a marked increase in p65 and pro-inflammatory cytokines expression in the cells co-transfected with wild-type miR-125b. A mutation in miR-125b seed region that alters the binding affinity of miR-125b with A20 resulted in a significant decline in the p65 and pro-inflammatory cytokine expression. We concluded from our findings that LPS-triggered activation of NFκB signaling is negatively regulated by A20 and subsequently helps in the resolution of inflammation under steady state. We also suggest that the increased miR-125b expression in UC patients might be responsible for the decreased A20 expression. Also, the miR-125b-mediated suppression of A20 expression also disturbs the resolution of inflammation to be carried out by A20. Therefore, downregulation of this key molecule might partially contribute to the UC pathogenesis and leads to chronic inflammation. Our study supports the earlier observations where A20 was shown to control inflammatory response mediated through inhibition of NFκB (27).

STAT3 was also identified as miR-125b target, and its expression levels were reduced in the colonic tissues of UC patients as compared to non-IBD controls, but the change in expression level was not significant even with a large sample size. Therefore, we did not pursue further validation for miR-125b and STAT3 through *in vitro* studies.

The qRT-PCR results indicated an inverse expression pattern for miR-223 and IKKα in UC patients with miR-223 being upregulated and IKKα getting downregulated. We further investigated whether the miR-223-targeted decrease in IKKα affects the NFκB-mediated inflammatory response through *in vitro* studies. Both p65 and pro-inflammatory cytokines (IL-8 and IL-1β) showed higher mRNA levels in the cells co-transfected with wild-type miR-223 as compared to the cells with empty pBABE-puro vector and IKKα-3′UTR. Again, the cells when co-transfected with the mutated form of miR-223 and IKKα-3′UTR showed decreased level of both p65 and pro-inflammatory cytokines. From these results, we concluded that the negative regulation of NFκB signaling is mediated by IKKα, thereby controlling the inflammatory response. These findings are consistent with the previous findings where the role of IKKα in resolving NFκB-mediated inflammatory response was explored (25, 43). Therefore, our study indicated that the significant upregulation of miR-223 in UC patients might contribute to UC pathogenesis by suppressing the expression of IKKα that has a critical role in controlling inflammation.

Lastly, we can conclude that miR-125b and miR-223 play an essential role in regulating the inflammatory response as they can efficiently target the key molecules of the NFκB signaling pathway. TRAF6 and A20, which are the key signaling molecules of NFκB signaling, bear binding sites for miR-125b in their 3′UTRs, and this results in negative regulation of TRAF6 and A20 expression by miR-125b. The increased pro-inflammatory response observed in TRAF6 absence in LPS-stimulated cells in our study highlights the additional regulatory role of TRAF6 as an adaptor molecule in NFκB signaling. We suggest that besides the E3 ubiquitin ligase activity, TRAF6 performs regulatory functions as well but the exact mechanism of this regulation and the targeted site for TRAF6 is still to be explored. A20 is very well-known as a negative feedback regulator of NFκB signaling where it rescues the cells from exaggerated pro-inflammatory response, thereby maintaining immune homeostasis. The presence of a miR-125b binding site within the A20 3′UTR results in higher pro-inflammatory response in LPS-stimulated HT29 cells. On the basis of these findings, we propose that miR-125b-mediated downregulation of TRAF6 and A20 in UC patients might be an additional factor for the chronic inflammation as the regulatory functions of A20 and IKKα are hampered by miR-125b, and they fail to restore the normal homeostasis. Another important finding from our study is the role of IKKα in regulating the inflammatory response generated through the canonical NFκB pathway, which is a key molecule involved in the non-canonical NFκB signaling. The decreased IKKα expression in UC patients and an increased pro-inflammatory response in LPS-stimulated cells in its absence indicate the possible role of IKKα in regulating inflammatory response in UC patients. Since both miR-125b and miR-223 are upregulated in UC patients and negatively regulate the gene involved in a major inflammatory pathway, we propose that these miRNAs could be considered as potential diagnostic biomarkers for UC to assess the disease development. In compliance with a previous study, we highlight the importance of miR-223 in UC pathogenesis, and higher expression of this microRNA has been reported not just from the colonic mucosal samples but also from the serum samples obtained from UC patients (44). Thus, our study further reiterates that miR-223 can be proposed as a promising biomarker for screening and diagnosis of UC. Through *in vitro* studies, we have investigated the probable role of miR-125b and miR-223 during inflammation. These findings can be explored in more detail through *in vivo* studies as UC is known to develop due to a complex interplay of microbial, genetic, and immune factors that cannot be reproduced successfully through *in vitro* studies.

## Data Availability Statement

Publicly available datasets were analyzed in this study. This data can be found here: GEO accession number GSE99632, https://www.ncbi.nlm.nih.gov/pubmed/28839432.

## Ethics Statement

The studies involving human participants were reviewed and approved by Institute Ethics Committee, All India Institute of Medical Sciences (Ref. No. T-290/23.06.2015, RT-7/27.01.2016), Institutional Ethics Review Board, JNU (IERB Ref. No. 2016/Student/93). The patients/participants provided their written informed consent to participate in this study.

## Author Contributions

SV and JP designed the study, analyzed the data, and prepared the manuscript. SV performed the experiments. JP and NP contributed the workspace, materials, reagents, analysis tools. VA provided the samples with clinical history.

### Conflict of Interest

The authors declare that the research was conducted in the absence of any commercial or financial relationships that could be construed as a potential conflict of interest.
